# White coat hypertension is another clinical characteristic of patients with inflammatory bowel disease: A cross-sectional study

**DOI:** 10.1097/MD.0000000000029722

**Published:** 2022-11-04

**Authors:** Vedran Premužić, Radovan Prijić, Mislav Jelaković, Željko Krznarić, Silvija Čuković-Čavka, Bojan Jelaković

**Affiliations:** a Department of Nephrology, Hypertension, Dialysis and Transplantation, ESH Excellence Center, University Hospital Center Zagreb, Zagreb, Croatia; b School of Medicine University of Zagreb, Zagreb, Croatia; c Department of Gastroenterology University Hospital Center Zagreb, Zagreb, Croatia.

**Keywords:** ambulatory blood pressure monitoring, arterial stiffness, cardiovascular risk, inflammatory bowel disease, pulse wave velocity, white coat hypertension

## Abstract

In this cross-sectional study, our aim was to analyze association of ambulatory blood pressure monitoring (ABPM) values with pulse wave velocity (PWV) in inflammatory bowel disease (IBD) patients as well as the prevalence and characteristics of white coat hypertension (WCH) in this group of patients with chronic inflammation and high prevalence of anxiety. We enrolled 120 consecutive IBD patients (77 Crohn´s disease; 43 ulcerative colitis) who were not treated with antihypertensive drugs without cardiovascular, cerebrovascular and renal morbidity. Office blood pressure, ABPM, and PWV were measured with Omrom M6, SpaceLab 90207, and Arteriograph, respectively. The prevalence of true normotension, sustained hypertension and WCH was analyzed in IBD patients. WCH was found in 27.5% patients. IBD-WCH patients had significantly lower prevalence of traditional risk factors than general WCH subjects. PWV and augmentation index (AIx) values were higher in WCH than in true normotensive patients. When adjusted for age and duration of IBD, only PWV was a positive predictor of WCH, and patients with higher PWV and longer disease duration had OR´s for WCH of 0.69 and 2.50, respectively. IBD patients had significantly higher prevalence of WCH and higher PWV values than healthy control patients. WCH is highly prevalent in IBD patients but IBD-WCH patients have lower frequency of traditional cardiovascular risk factors than general WCH population. Our results suggest that WCH could be considered as another clinical characteristic of IBD which is associated with increased arterial stiffness and those patients should be monitored more closely.

## 1. Introduction

Inflammatory bowel disease (IBD), an immune-mediated inflammatory disease is associated with high cardiovascular (CV) morbidity and mortality despite lower prevalence of traditional CV risk factors.^[1–5]^ Chronic inflammation was proposed as one of explanations for this, so-called “IBD paradox” and duration of IBD and disease activity were recognized as important CV risk factors in IBD.^[3,5–8]^ Higher pulse wave velocity (PWV) and augmentation index (AIx) were found in IBD patients and increased arterial stiffness was proposed to be linked to higher CV risk in IBD patients.^[9–15]^ Observed increased arterial stiffness in IBD patients without presence and cumulative effect of other CV risk factors further contributes to the “IBD paradox” and some authors suggested that increased arterial stiffness in IBD might be a link between chronic inflammation and increased CV risk in IBD patients.^[5,9,14]^ Zanoli et al proposed that different mechanisms lead to arterial stiffness in IBD compared to chronic kidney disease (CKD) suggesting chronic inflammation, but not aging and blood pressure (BP), as the most important risk factor for increased PWV in IBD patients which is in line with observations that disease duration is strongly associated with arterial stiffness.^[13–21]^ Majority of authors agree that higher anxiety in general population is related to higher risk of white coat hypertension (WCH)^[22–24]^ which can affect PWV.^[25–29]^ Stress was found to have a role in pathogenesis and prognosis of IBD^[30]^ while IBD is associated higher incidence of personality disorders.^[31]^ Some would expect that due to the fact that the prevalence of anxiety is 29% to 35% during remission and even 80% during relapse of IBD^[31,32]^ there was a reported association with WCH and PWV in this group of patients. Surprisingly, searching the literature we found only one paper where BP in IBD patients was measured using ambulatory blood pressure monitoring (ABPM) but those authors did not analyze association of ABPM values with arterial stiffness.^[33]^ Therefore, our aim was to analyze association of ABPM values with PWV in IBD patients. Due to the fact that these patients are characterized with chronic inflammation we have also analyzed the prevalence and characteristics of WCH in IBD.

## 2. Methods

In this study 120 consecutive IBD patients (77 patients with Crohn´s disease and 43 patients with ulcerative colitis) were enrolled in period from January 2015 to January 2018. They were recruited at routine checkups and they have signed the informed consent in order to participate in the study. Exclusion criteria were: antihypertensive drugs, limb amputees, history of mental illness, atrial fibrillation or other chronic arrhythmias, NYHA III-IV stage congestive heart failure, decompensated liver disease or ascites, history of stroke, transient ischemic attack, myocardial infarction or malignant disease. The detailed medical history of the patients was collected and complete physical examination was conducted. Office blood pressure was measured using Omrom M6 device and appropriate cuff according to the European Society of Cardiology/European Society of Hypertension guidelines.^[34]^ Measurement was done in a dedicated room in a recumbent position after 15 minutes rest and office blood pressure values were determined as a mean of three measurements. In all patients following laboratory data were collected: complete blood count, C-reactive protein, fibrinogen, serum glucose, cholesterol, triglycerides, sodium, potassium, calcium, phosphorus, serum creatinine, bilirubin, liver transaminases, gamma-glutamyl transferase, alkaline phosphatase, hemoglobin A1c and 24 hour albuminuria. Glomerular filtration rate was estimated using CKD EPI equation and CKD was considered if estimated glomerular filtration rate (eGFR) was <60 mL/min/1.73 m^2^. ABPM was performed using SpaceLab 90207 (Washington, DC) on regular working day. The monitor was programmed to perform readings at 15-minutes intervals between 6 am and 10 pm (day-time), and at 30-minutes intervals between 10 pm and 6 am (nighttime). The noninvasive measurement of pulse wave velocity (PWV), as an index of arterial stiffness, was performed in IBD group of patients using validated, noninvasive device (Tensiomed Arteriograph device [Medexpert Ltd., Budapest, Hungary]) which uses oscillometric method simultaneously measuring brachial blood pressure, PWV and AIx.^[35]^ The central blood pressure was determined with the same device. Measurement was done in a dedicated room in a recumbent position after 15 minutes rest. PWV and AIx were determined as a mean of three measurements. True normotension was defined as office BP < 140/90 mm Hg and awake daytime ABPM < 135/85 mm Hg, sustained hypertension was defined as office BP ≥ 140/90 mm Hg and awake daytime ABPM > 135/85 mm Hg, and WCH was diagnosed if office BP > 140/90 mm Hg and awake daytime ABPM < 135/85 mm Hg. The patients with nighttime ABPM drop <10%, 10% to 20%, and more than 20% were defined as non-dippers, dippers and extreme dippers. Average PWV of 7.8 ± 2.2 m/s was reported in patients with systemic lupus thus we determined a cutoff value for increased arterial stiffness of PWV > 8 m/s.^[36]^ CKD was defined as eGFR < 60 mL/min/1.73 m^2^, albuminuria was defined as 24 hour albuminuria > 30 mg/d. Left ventricular hypertrophy was defined using voltage ECG Sokolow-Lyon Criteria: if the sum of S wave in V1 plus the R wave in V5 or V6 > 35 mm. The activity of Chron´s disease and ulcerative colitis were assessed by Harvey–Bradshaw index and Truelove and Witts’ criteria, respectively.^[37,38]^ We have enrolled 90 healthy age-sex matched and untreated control subjects which were recruited at family physician office during general medical examination to compare the incidence of WCH and PWV values with IBD group of patients. The protocol was approved by the hospital ethics committee (University Hospital Center Zagreb, number 02/21 AG), in accordance with the Helsinki Declaration, and all participants gave written informed consent.

Statistical analysis was performed using SPSS version 23.0 (IBM Corp., Armonk, NY). Normality of data distribution was tested using Kolmogorov–Smirnov test. Preliminary analyses were performed to ensure no violation of the assumptions of normality, linearity and homoscedasticity. Categorical data were expressed as numbers and frequencies. Correlations were obtained using Pearson’s test for normally distributed variables and Spearman rank correlation for non-normally distributed variables. Normally distributed variables were presented as means ± standard deviations and Student’s *t* test for independent samples was used for comparisons between two groups. Non-normally distributed data was presented as median and interquartile range and Mann–Whitney *U* test was used in comparison between two groups. Categorical variables were compared using χ^2^ test. Binary logistic regression was made to determine associations of elevated PWV levels. Multiple linear regression was used to explore the influence of different variables on PWV levels. A *P* value < .05 (two-sided tests) was considered significant.

## 3. Results

### 3.1. Characteristics of whole group of IBD patients

Mean age of our group of 120 IBD patients was 37.1 ± 4.2 years. Demographic, clinical biochemical and hemodynamic data of all enrolled patients are shown in Table 1. Diabetes was diagnosed in seven patients (5.8%) and 39 patients (32.5%) had hypertension based on office BP measurements. Dyslipidemia was observed in 12 (10%) patients while LVH, CKD (eGFR < 60 mL/min/1.73 m^2^) and 24-hour albuminuria were found in 1 (0.83%), 2 (1.67%), and 18 (15%) patients, respectively.

**Table 1:** Demographic, clinical, biochemical, and hemodinamic data on the whole IBD group.

	Whole group
Age (yr)	37.1 ± 4.2
Men (%)	58.3
Body mass index (kg/m^2^)	24.7 ± 3.4
Duration of disease (mo)	127.0 ± 10.4
IBD type N (%)	
Ulcerative colitis	43 (35.8)
Chron’s disease	77 (64.2)
Disease activity N (%)	
Remission	7 (5.8)
Mild disease	33 (27.5)
Moderate disease	36 (30.0)
Severe disease	44 (36.7)
Disease activity score	
Harvey–Bradshaw index score (N = 77)	2.13 ± 0.7
Truelove & Witts score (N = 43)	1.98 ± 0.4
Immunosuppression Yes (%)	78.6
Biologic treatment Yes (%)	51.7
Diabetes Yes (%)	5.8
Smokers (%)	12.5
Hemoglobin (g/L)	126.5 ± 18.0
White blood count (10^9^/L)	10.2 ± 2.1
C-reactive protein (mg/L)	14.5 ± 1.7
Total serum cholesterol (mmol/L)	3.72 ± 1.1
HDL-cholesterol (mmol/L)	1.15 ± 0.4
LDL-cholesterol (mmol/L)	1.95 ± 1.1
Serum triglycerides (mmol/L)	1.36 ± 0.9
Serum creatinine (µmol/L)	74.1 ± 16.6
eGFR (mL/min/1.73 m^2^)	104.5 ± 10.2
24 h albuminuria (mg/dU)	19.9 ± 3.9
Albuminuria (%)	15
Left ventricular hypertrophy (%)	0.83
Dyslipidemia (%)	10
Chronic kidney disease N (%)	1.67
Office SBP (mm Hg)	124.5 ± 16.3
Office DBP (mm Hg)	73.4 ± 11.9
Office heart rate (bpm)	76 (59–115)
ABPM 24 h SBP (mm Hg)	113.7 ± 10.5
ABPM 24 h DBP (mm Hg)	71.9 ± 7.5
ABPM 24 h HR (mm Hg)	72 (56–109)
ABPM day SBP (mm Hg)	115.6 ± 11.2
ABPM day DBP (mm Hg)	73.8 ± 7.8
ABPM day HR (mm Hg)	74 (58–112)
ABPM night SBP (mm Hg)	108.1 ± 10.1
ABPM night DBP (mm Hg)	66.7 ± 8.1
ABPM night HR (mm Hg)	71 (52–106)
Dipper (%)	52.5
Non dipper (%)	41.7
Reverse dipper (%)	0
Extreme dipper (%)	5.8
True hypertension N (%)	1 (0.83)
Masked hypertension N (%)	1 (0.83)
White coat hypertension N (%)	33 (27.5)
True normotension N (%)	85 (71.6)
Central systolic blood pressure (mm Hg)	113.9 ± 18.4
Central pulse pressure (mm Hg)	40.6 ± 10.3
Pulse wave velocity (m/s)	8.9 ± 1.4
Augmentation index (%)	14.8 ± 1.5

Results are shown as mean ± SD or median (interquartile range).

ABPM = ambulatory blood pressure monitoring, DBP = diastolic blood pressure, GFR = glomerular filtration rate, HR = heart rate, IBD = inflammatory bowel disease, SBP = systolic blood pressure.

### 3.2. The association of large artery stiffness with IBD

Interestingly, PWV above the cut off value was found in 54 (45%) of patients. PWV was positively correlated with age (*r* = 0.591; *P* < .001), duration of IBD (*r* = 0.328; *P* < .001) and heart rate (*r* = 0.345, *P* < .001) while AIx was positively correlated with age (*r* = 0.299; *P* < .001), duration of IBD (*r* = 0.582; *P* < .01), and negatively with heart rate (*r* = −0.285, *P* = .002).

In the whole group, in the linear regression model age and duration of IBD were only and positive predictors of higher PWV (β = 0.468 and β = 0.057, all *P* < .05) while we have not found association with dyslipidemia, any of BP values and diabetes. When adjusted for age and duration of IBD, positive predictors of higher PWV were disease activity and steroid therapy (β = 0.333, β = 0.250 and β = 0.189, all *P* < .05). On logistic regression older patients and patients with longer duration of IBD had OR´s for PWV > 8 m/s of 0.94 [CI 0.89, 0.99] and 0.99 [CI 0.98, 1.00]. When adjusted for age, only duration of IBD had significant OR for PWV > 8 m/s of 0.99 [CI 0.98, 0.99].

### 3.3. ABPM values, BP patterns and determinants of WCH in IBD patients

After analyses of ABPM values WCH was found in 33 subjects (27.5%) and true hypertension and masked hypertension were detected in one patient respectively; those two patients were excluded from further analyses. Fifty patients (41.7%) with IBD were non-dippers.

In linear regression model duration of IBD and PWV were only and positive predictors of WCH (β = 0.442 and β = 0.312, all *P* < .05) while we have not found association with age, any of BP values and diabetes. When adjusted for age and duration of IBD, only PWV was a positive predictor of WCH (β = 0.177, *P* < .05). Patients with longer disease duration had OR´s for WCH of 2.50 (CI 1.08, 5.77), respectively.

### 3.4. Differences between IBD patients with WCH and true normotension

We failed to find differences in age, gender, smoking status, duration of IBD, biochemical data, immunosuppressive and biologic treatment between WCH and true normotensive patients (Table 2). Furthermore, there were no significant differences in disease activity score between WCH and true normotensive patients. Regarding ABPM data we have not found differences in day-time and nigh-time systolic and diastolic BP and heart rate values between WCH and true normotensive patients while WCH patients had significantly higher office SBP and DBP values than true normotensive patients as expected (Table 3). Additionally, we failed to find differences in dipping pattern between two groups. PWV and AIx values were higher in WCH than in true normotensive patients, although differences were statistically insignificant. However, significantly more patients with WCH had PWV above the cutoff value than true normotensive subjects (*P* < .001). Finally, patients with WCH had significantly higher values of central systolic BP (*P* < .01) and central pulse pressure (*P* < .001).

**Table 2:** Demographic, clinical and biochemical data on inflammatory bowel disease patients with white coat hypertension and true normotension.

	White coat hypertension (N = 33)	True normotension (N = 85)	*P*
Age (yr)	38.8 ± 4.8	36.3 ± 3.0	.43
Men (%)	54.5	52 (59.7)	.84
Smokers (%)	9.0	12 (13.7)	.48
Body mass index (kg/m^2^)	24.1 ± 3.2	25.2 ± 3.9	.56
Prior diabetes Yes (%)	3.0	6 (6.8)	.39
Duration of IBD (mo)	139.1 ± 10.1	122.4 ± 9.5	.48
IBD type N (%)			
Ulcerative colitis	20 (60.6)	57 (65.5)	.62
Chron’s disease	13 (39.4)	30 (34.5)	
Immunosuppression Yes (%)	20 (60.6)	50 (57.4)	.51
Biologic treatment Yes (%)	15 (45.5)	31 (35.6)	.22
IBD activity N (%)			
Remission	1 (3.0)	6 (6.8)	.39
Mild disease	12 (36.4)	21 (24.1)	.17
Moderate disease	11 (33.3)	25 (28.7)	.55
Severe disease	12 (36.3)	32 (36.7)	.88
IBD activity score			
Harvey–Bradshaw index score	2.00 ± 0.4	2.20 ± 0.8	.43
Truelove & Witts score	2.14 ± 0.8	1.94 ± 0.4	.61
White blood count (10^9^/L)	8.0 ± 2.5	11.1 ± 2.7	.29
C-reactive protein (mg/L)	13.3 ± 1.1	14.9 ± 1.9	.66
Serum cholesterol (mmol/L)	3.69 ± 1.0	3.70 ± 1.1	.84
HDL-cholesterol (mmol/L)	1.19 ± 0.4	1.12 ± 0.5	.46
LDL-cholesterol (mmol/L)	2.10 ± 1.3	1.90 ± 0.9	.48
Serum triglycerides (mmol/L)	1.23 ± 0.7	1.41 ± 1.0	.21
Serum creatinine (µmol/L)	74.6 ± 18.6	73.9 ± 13.5	.86
eGFR (mL/min/1.73 m^2^)	110.2 ± 9.9	113.3 ± 14.7	.77
24 h albuminuria (mg/dU)	26.7 ± 5.1	17.3 ± 4.2	.27
Dyslipidemia (%)	9.1	10.6	.81
Chronic kidney disease (%)	0	2.3	.37
Albuminuria (%)	9.1	17.6	.25
Left ventricular hypertrophy (%)	0	1.1	.83

Results are shown as mean ± SD or median (interquartile range).

GFR = glomerular filtration rate, HDL = high-density lipoprotein, IBD = inflammatory bowel disease, LDL = low-density lipoprotein.

**Table 3:** Blood pressure values and arterial stiffness markers on inflammatory bowel disease patients with white coat hypertension and true normotension.

	White coat hypertension (N = 33)	True normotension (N = 85)	*P*
Office SBP (mm Hg)	140.1 ± 23.1	118.6 ± 12.2	<.001
Office DBP (mm Hg)	84.6 ± 15.4	69.2 ± 10.1	<.001
Office heart rate (bpm)	78 (60-116)	76 (57-112)	.84
ABPM 24 h SBP (mm Hg)	114.2 ± 12.9	112.1 ± 12.3	.39
ABPM 24 h DBP (mm Hg)	72.4 ± 12.2	70.7 ± 11.1	.37
ABPM 24 h HR (mm Hg)	78 (57-112)	75 (55-111)	.79
ABPM day SBP (mm Hg)	116.1 ± 13.5	114.2 ± 12.9	.47
ABPM day DBP (mm Hg)	74.1 ± 13.7	72.9 ± 11.8	.37
ABPM day HR (mm Hg)	79 (59-112)	78 (60-113)	.54
ABPM night SBP (mm Hg)	108.6 ± 10.7	106.6 ± 10.1	.37
ABPM night DBP (mm Hg)	67.3 ± 10.4	65.3 ± 9.7	.28
ABPM night HR (mm Hg)	76 (57-110)	72 (58-109)	.54
Dipper (%)	57.6	51.8	
Non dipper (%)	39.4	43.5	.57
Reverse dipper (%)	0	0	.68
Extreme dipper (%)	3.0	4.7	.69
Central SBP (mm Hg)	130.5 ± 20.9	107.6 ± 13.5	<.001
Central pulse pressure (mm Hg)	46.1 ± 12.3	38.5 ± 7.1	<.01
Pulse wave velocity (m/s)	8.5 ± 1.6	7.8 ± 0.7	.10
Pulse wave velocity > 8 m/s (%)	84.8	30.6	<.001
Augmentation index	17.9 ± 1.6	13.6 ± 1.2	.22

Results are shown as mean ± SD or median (interquartile range), categorical variables were compared using χ^2^ test.

ABPM = ambulatory blood pressure monitoring, DBP = diastolic blood pressure, HR = heart rate, SBP = systolic blood pressure.

### 3.5. Differences between IBD patients and healthy control patients

We failed to find differences in age and gender between IBD and healthy control patients (Table 4). Prevalence of WCH was significantly higher in the IBD group than in the control group (27.5% vs 13.3%). Average 24 hour, day-time and nigh-time systolic BP values were significantly higher in control group than in IBD patients. As expected, IBD group had significantly higher prevalence of patients with non-dipping and extreme dipping pattern as well as higher values of PWV and AIx than control group. Significantly more patients with IBD had PWV above the cutoff value than true normotensive subjects (*P* < .01). Finally, patients with IBD had significantly higher values of central systolic BP (*P* = .01).

**Table 4:** Demographic, blood pressure values and arterial stiffness markers data between inflammatory bowel disease patients and healthy controls.

	IBD (N = 120)	Healthy controls (N = 90)	*P*
Age (yr)	37.1 ± 4.2	39.3 ± 4.7	.08
Men (%)	58.3	61.1	.68
Body mass index (kg/m^2^)	24.7 ± 3.4	24.1 ± 3.2	.82
Office SBP (mm Hg)	124.5 ± 16.3	125.1 ± 16.2	.77
Office DBP (mm Hg)	73.4 ± 11.9	75.6 ± 12.1	.15
Office heart rate (bpm)	76 (59–115)	75 (56–111)	.34
ABPM 24 h SBP (mm Hg)	113.7 ± 10.5	122.4 ± 18.1	<.001
ABPM 24 h DBP (mm Hg)	71.9 ± 7.5	71.4 ± 7.2	.64
ABPM 24 h HR (mm Hg)	72 (56–109)	74 (56–112)	.81
ABPM day SBP (mm Hg)	115.6 ± 11.2	128.2 ± 14.3	<.001
ABPM day DBP (mm Hg)	73.8 ± 7.8	76.1 ± 12.4	.07
ABPM day HR (mm Hg)	74 (58-112)	76 (59-114)	.67
ABPM night SBP (mm Hg)	108.1 ± 10.1	115.3 ± 14.2	<.001
ABPM night DBP (mm Hg)	66.7 ± 8.1	66.5 ± 9.9	.86
ABPM night HR (mm Hg)	71 (52–106)	71 (53–107)	.89
Dipper (%)	52.5	63.1	
Non dipper (%)	41.7	18.5	.03
Reverse dipper (%)	0	0	<.001
Extreme dipper (%)	5.8	0	<.001
White coat hypertension (%)	27.5	13.3	<.01
Central SBP (mm Hg)	113.9 ± 18.4	122.1 ± 25.3	.01
Central pulse pressure (mm Hg)	40.6 ± 10.3	39.5 ± 8.3	.12
Pulse wave velocity (m/s)	8.9 ± 1.4	7.6 ± 0.7	<.001
Pulse wave velocity > 8 m/s (%)	45.0	26.6	<.01
Augmentation index	14.8 ± 1.5	22.4 ± 1.2	<.001

ABPM = ambulatory blood pressure monitoring, DBP = diastolic blood pressure, GFR = glomerular filtration rate, HR = heart rate, IBD = inflammatory bowel disease, SBP = systolic blood pressure.

## 4. Discussion

In our group of IBD patients we found high prevalence of WCH (27.5%) being higher than prevalence observed by other authors in general population using the same diagnostic criteria (9–17.9%)^[29,39–41]^ as well as in our control group of healthy subjects (13.3%) where we have find significantly lower prevalence of patients with non-dipping and extreme dipping pattern than in IBD group. We found our IBD-WCH patients to be significantly younger than non-IBD WCH subjects included in other studies (38.8 vs 52.3–58.8 years of age) with slightly lower female prevalence compared to others. Proportion of smokers in our WCH-IBD group was the same as Scuteri et al^[29]^ observed in non-IBD WCH but significantly lower than other authors detected in general WCH subjects.^[25,27–29]^ Prevalence of diabetes was lower in our IBD-WCH patients compared to non-IBD-WCH subjects and values of lipids were substantially lower in IBD-WCH patients than values observed by other authors in non-IBD-WCH subjects.^[7–12]^ None of our IBD-WCH patients had dyslipidemia or eGFR < 60 mL/min/1.73 m^2^ and albuminuria was detected in 9.1 % patients being similar to 8.5% observed in non-IBD WCH subjects enrolled in the Spanish ABPM registry.^[42]^ However, there we no difference in albuminuria between IBD-WCH and true normotensive IBD patients. To summarize, IBD-WCH patients had significantly lower prevalence of traditional CV risk factors than non-IBD-WCH subjects who have increased risk of CV events and all-cause mortality, risk being intermediate between normotension and sustained hypertension.^[11,28,29,40–51]^ Our results are in agreement with general observation that IBD is associated with lower frequency of traditional CV risk factors. Furthermore, in our group of IBD patients we failed to find differences in age, gender, smoking habits, diabetes, lipid parameters and eGFR between WCH and true normotensive patients. IBD-WCH patients had higher nighttime heart rate than non-WCH IBD patient, although difference did not reach statistical significance. No significant differences in other ABPM parameters were observed between IBD-WCH patients and true normotensive IBD patients. Pourafkari et al analyzed ABPM characteristics in a group of IBD patients, mostly with ulcerative colitis, and found high prevalence of masked hypertension (24%) what is different from our results.^[33]^ In their study office BP ≥ 140/90 mm Hg was an exclusion criteria and because of that 6 out of 63 patients were not enrolled.^[25]^ Thus, it could be speculated that the prevalence of WCH was significantly lower in their IBD patients than we found in our group. Difference in ABPM phenotype between results obtained by Pourafkari et al^[33,51,52]^ and us could be explained with differences in inclusion/exclusion criteria, characteristics between two groups of patients but also could reflect moderate reproducibility of WCH and masked hypertension. Considering lower prevalence of traditional CV risk factors in IBD-WCH patients one could argue that WCH in IBD is not associated with higher CV risk and consecutively that high prevalence of WCH could not contribute to the observed higher CV risk in IBD patients.

It was reported that WCH is associated with early vascular aging and two meta-analyses confirmed that subjects with WCH, both treated and untreated, have increased cf-PWV compared to the normotensive subjects^[27,29,37,53,54]^ which was in line with our results. It was proposed that observed higher CV risk in WCH partly could be explained with higher PWV, i.e., higher arterial stiffness. The higher values of PWV in WCH were reported to be related to observed higher 24 hour ABPM values than in normotensives.^[39]^ However, in WCH subjects Andrikou et al^[55]^ found association of CRP with PWV suggesting that inflammation might contribute to increased arterial stiffness. Higher values of PWV observed in patients with IBD also were explained with chronic inflammation. It was shown that effective control of chronic inflammation with anti-tumor necrosis factor alpha in IBD patients reduces aortic PWV which may reduce CV risk.^[17,20]^ Our data are in agreement with majority of authors who found increased arterial stiffness in IBD patients and confirmed with higher values of PWV and AIx than in control group. Average PWV in our IBD group was higher than PWV determined in the age and sex-matched subjects from the general population, and PWV above the cutoff value of 8 m/s was found in significantly higher percentage of patients when compared to healthy controls. Average PWV in our group of IBD patients who had on average 37.1 years of age was 8.9 m/s compared to 7.6 m/s, 6.7 m/s, and 7.29 m/s from the age-matched subjects from our healthy control group, subjects from general population or from general population using Arteriograph as we did.^[56,57]^ Importantly, our results are in line with results obtained by Ozturk et al who also used Arteriograph in IBD patients who were on average 7 years younger than our patients (Crohn disease 8.13 ± 1.61; ulcerative colitis 8.16 ± 1.74; healthy subjects 6.85 ± 0.95).^[10]^ We found that duration of IBD and disease activity, but not aging or BP values, are positive predictors of higher PWV. Our data are in concordance with observations from Zanoli et al and results published by Korkmaz et al^[16,18]^ who found that duration of IBD positively correlate with 24-hour PWV. Based on these results it could be proposed that long lasting chronic inflammation is related to higher arterial stiffness in our IBD patients. We did not find association of PWV with CRP what is agreement with recent individual participant data meta-analyses.^[20,21]^ Our IBD-WCH patients had higher, although statistically insignificant, values of PWV and AIx than true normotensive IBD patients and statistically higher values of PWV and AIx than healthy controls. There were significantly more patients with PWV above the cut off value in IBD-WCH compared to true normotensive IBD patients and healthy control patients. When adjusted to age and duration of IBD, PWV was only positive predictor of WCH. Longer duration of chronic inflammation is associated with higher PWV but as reported by other authors also with higher incidence of anxiety which could explain higher occurrence of WCH in IBD.^[30–33]^ In addition, higher PWV in patients with WCH could be result of periodically increased BP and functional increase of arterial stiffness.

Our study has several limitations. First, this small cross-sectional study is the report from one center and our results should be confirmed by other authors. Secondly, ABPM was performed only once and we did not analyze occurrence of anxiety. Both parameters would substantially improve our results. Furthermore, we analyzed association of ABPM values with cf PWV but valuable data could be obtained if we had additionally measured brachial PWV. Our study has several important strengths. According to our knowledge this is the first report on ABPM and PWV in IBD (both Chron and ulcerative colitis) and the first report in these patients on association of BP measured using ABPM and PWV. Furthermore, this is the first study on characteristics of WCH in IBD.

According to our results, neither office BP nor ABPM is related to increased PWV in IBD patients, and disease duration and disease activity, i.e. chronic inflammation, are the most important predictors of PWV in IBD. IBD-WCH have lower frequency of traditional CV risk factors than general WCH population. However, increased PWV and higher central systolic BP and central pulse pressure can increase CV risk indicating that WCH in IBD is not an innocent condition. ABPM, central BP and PWV measurements should be conducted in all IBD patients. Longitudinal studies on the effect of WCH on arterial stiffness and its association with CV risk in IBD are warranted.

## Acknowledgments

All authors have read and approved the submission of the manuscript; the material is original research. Authors had no writing assistance.

## Author contributions

**Conceptualization:** Vedran Premužić, Radovan Prijić.

**Investigation:** Radovan Prijić.

**Methodology:** Vedran Premužić, Bojan Jelaković.

**Supervision:** Željko Krznarić, Bojan Jelaković.

**Validation:** Silvija Čuković-Čavka, Bojan Jelaković.

**Writing – original draft:** Vedran Premužić.

**Writing – review & editing:** Mislav Jelaković, Bojan Jelaković.
